# Systemic Sclerosis Presenting With Chronic Atrophic Gastritis and Retinal Artery Occlusion: A Case Report

**DOI:** 10.7759/cureus.111270

**Published:** 2026-06-22

**Authors:** Kristine Gvinepadze, Marika Toidze

**Affiliations:** 1 Rheumatology, Caucasus Medical Center, Tbilisi, GEO

**Keywords:** atrophic gastritis, megaloblastic anemia, retinal artery occlusion, systemic sclerosis, vitamin b12 deficiency

## Abstract

Systemic sclerosis (SSc), also known as scleroderma, is a rare autoimmune disease characterized by fibroproliferative changes, vascular dysfunction, and small-vessel proliferation. It can affect multiple organ systems, including the gastrointestinal tract, skin, heart, eyes, kidneys, and lungs.

We report a case of a 67-year-old woman with systemic sclerosis who tested negative for specific antibodies and simultaneously developed two rare complications: autoimmune atrophic gastritis resulting in megaloblastic anemia and acute retinal artery occlusion. Notably, she presented with megaloblastic anemia despite normal serum vitamin B12 levels, consistent with early deficiency due to parietal cell destruction and reduced intrinsic factor production.

This case emphasizes the importance of recognizing uncommon gastrointestinal and ocular manifestations in systemic sclerosis, as timely diagnosis and management are essential to prevent serious complications such as severe anemia and permanent vision loss.

## Introduction

Systemic sclerosis (SSc) is a rare autoimmune connective tissue disease that alters both innate and adaptive immune systems. It occurs more frequently in females and has three main subtypes: limited cutaneous systemic sclerosis (CREST syndrome), diffuse cutaneous systemic sclerosis, and systemic sclerosis sine scleroderma [1].

Chronic atrophic gastritis is an uncommon manifestation in systemic sclerosis, occurring in 0.3%-2.7% of cases, typically with a male predominance [2-5]. Due to its nonspecific gastrointestinal symptoms, it is frequently overlooked or misdiagnosed. Timely diagnosis is crucial to prevent serious complications such as malabsorption, vitamin B12 deficiency, and gastric malignancy [2-6]. The esophagus is the most commonly affected gastrointestinal site (30-75%) [6]. The diagnostic test of choice is gastric endoscopy with biopsy, which reveals diffuse infiltration of lymphocytes and plasma cells in the deep mucosal layer. As lymphocytic infiltration progresses, the fundic glands atrophy and eventually disappear, resulting in marked mucosal atrophy [2, 3, 5].

Ocular manifestations of systemic sclerosis can be divided into anterior and posterior segment involvement. Posterior segment involvement primarily affects the retina, optic nerve, and choroid [7, 8]. Although rare, acute retinal artery occlusion can occur, causing acute, painless, unilateral vision loss [7-9]. Differentiating the etiology of vision loss is essential, as hypertension due to scleroderma renal crisis is another important manifestation of the disease [7]. The retina is relatively protected from vasculopathy and fibrosis in systemic sclerosis owing to its immunologically privileged microcirculation, absence of resident fibroblasts, and limited adrenergic vasomotor innervation [8]. Common anterior segment changes include eyelid skin remodeling, dry eye syndrome, and conjunctival abnormalities [10].

Environmental factors, such as infectious agents, microbiome alterations, occupational exposures, dietary habits, lifestyle factors, and certain medications, are considered important triggers. These exposures can induce cell type-specific, stable, and heritable epigenetic modifications [6].

## Case presentation

A 67-year-old female patient presented to a rheumatologist with longstanding progressive arthritis (joint pain), epigastric pain, noticeable mood instability, and sudden painless unilateral vision loss. She could not recall the onset of her symptoms or which joint was affected first. She denied constitutional or other symptoms, such as weight loss, fever, other skin changes, generalized weakness, or fatigue.

Subsequently, she developed worsening epigastric and upper abdominal pain along with emotional instability. She had experienced Raynaud’s phenomenon since adulthood, although it had never been formally diagnosed. Her only known prior diagnosis was osteoporosis, for which she was taking ibandronic acid. She denied any drug allergies. The patient is a nonsmoker, and she does not have any trauma history.

On physical examination, the patient had puffy fingers, proximal myopathy in the upper extremities, and restricted range of motion in the left shoulder. The remainder of the physical examination was unremarkable, with no skin thickening, digital ulcers, or telangiectasia noted. The patient appeared quite agitated.

**Figure 1:**
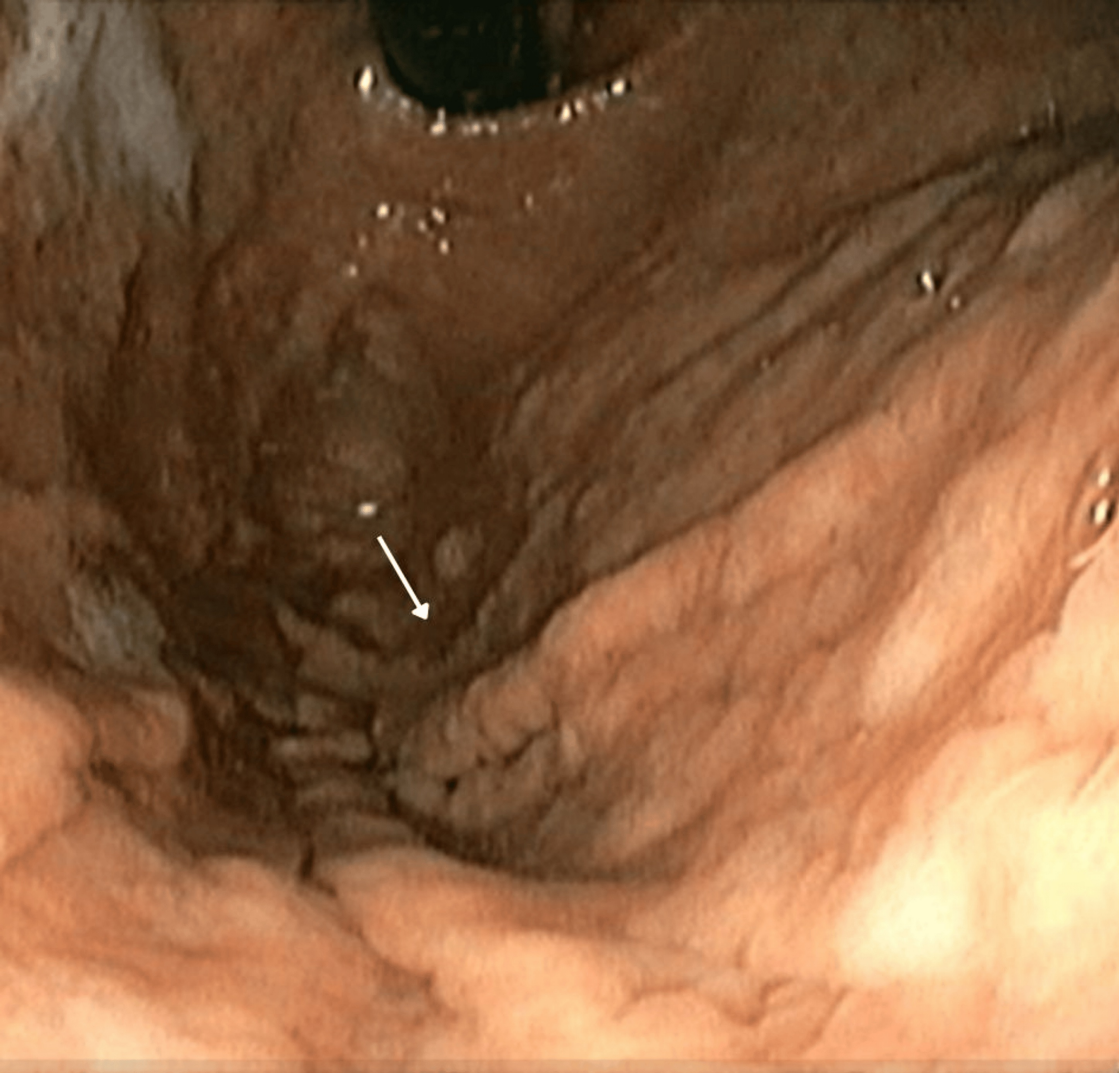
Upper Endoscopy Image Esophagogastroduodenoscopy showing the pyloric/antral region of the stomach. There are thickened, inflamed mucosal folds with patchy pallor, suggestive of focal atrophy. The overall appearance is consistent with chronic gastritis with active inflammation and areas of mucosal atrophy (white arrow).

**Figure 2:**
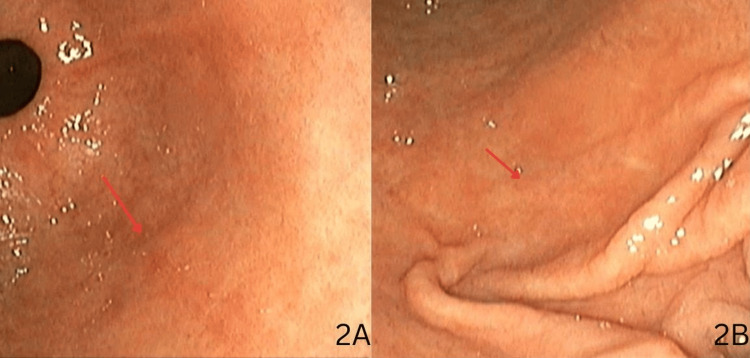
upper endoscopy images Hyperemic atrophic gastric mucosal surface showing White Globe Appearance (WGA) (red arrows). WGA, commonly observed in atrophic gastritis, represents cystic dilatation of gastric glands containing intraglandular debris or necrotic material.

**Figure 3:**
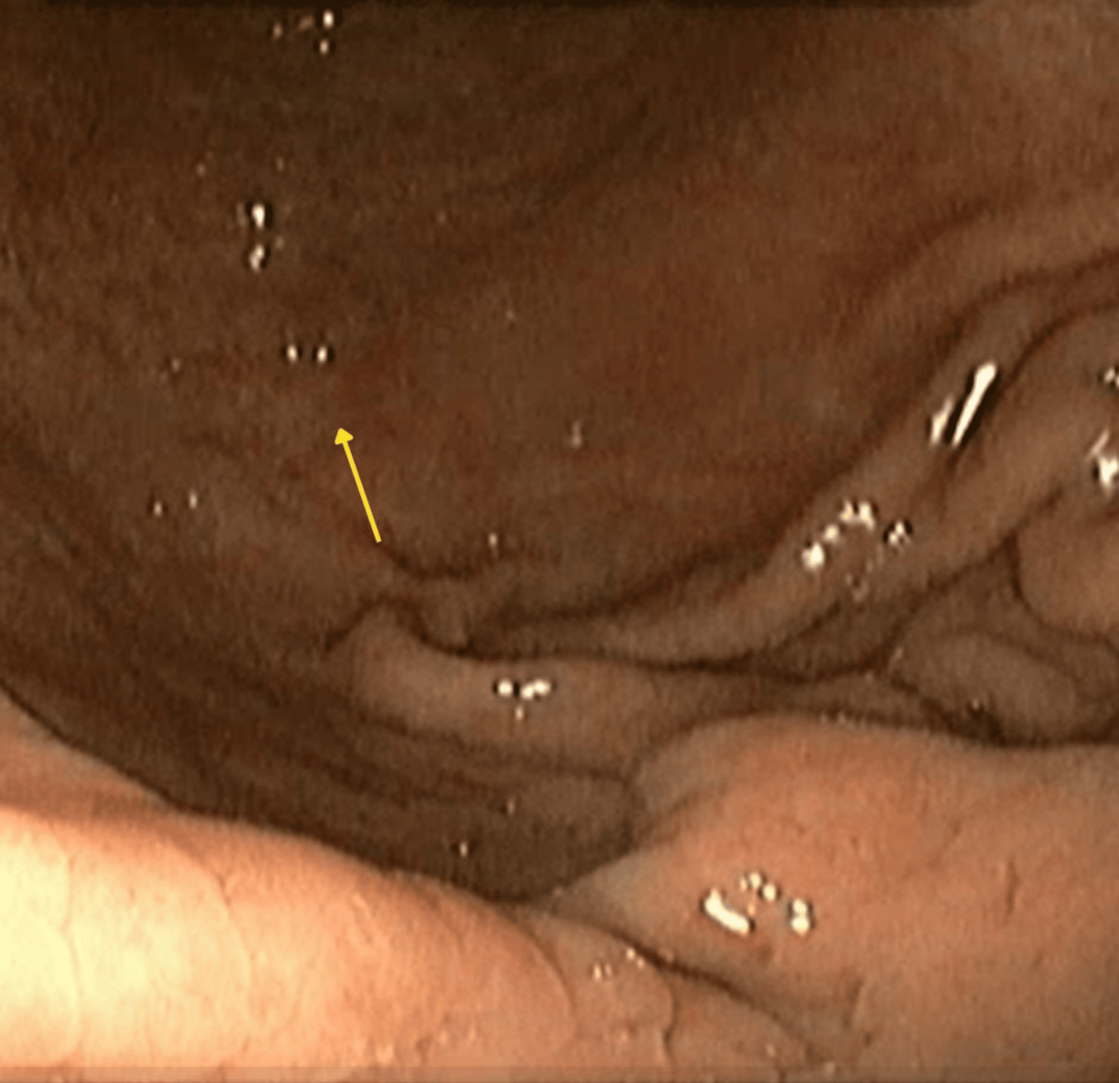
Upper Endoscopy Image Close-up endoscopic image of the gastric mucosa showing atrophic changes with hyperemia and multiple White Globe Appearances (WGAs) (yellow arrows). This finding is typical of atrophic gastritis and corresponds to cystic dilatation of gastric glands with intraglandular debris or necrotic material.

To evaluate for autoimmune diseases and assess her overall health status, the following laboratory tests were requested: complete blood count, antinuclear antibodies (ANA) panel, serum creatinine, thyroid-stimulating hormone (TSH), vitamin D levels, and C-reactive protein (CRP). Results are shown below. Given the patient’s gastric symptoms, endoscopy with biopsy was performed for further evaluation. Laboratory investigations revealed megaloblastic anemia accompanied by multiple vitamin and mineral deficiencies (Table 1), elevated inflammatory markers (CRP and ESR) (Table 1), and a positive ANA profile (anti-Ro52 and anti-SS-A antibodies) consistent with systemic sclerosis (Table 2). Upper gastrointestinal endoscopy with biopsy demonstrated hyperemic gastric mucosa with atrophic changes consistent with chronic atrophic gastritis (Figures 1, 2, 3), while *Helicobacter pylori *antigen testing was negative.

**Table 1:** Laboratory Results (December 2, 2025) RBC: red blood cell count; HGB: hemoglobin; HCT: hematocrit; MCV: mean corpuscular volume; MCH: mean corpuscular hemoglobin; MCHC: mean corpuscular hemoglobin concentration; PLT: platelet count; WBC: white blood cell count; ESR: erythrocyte sedimentation rate; TSH: thyroid-stimulating hormone; CRP: C-reactive protein

Parameter	Result (Normal Range)
Vitamin B12 serum level	718 pg/mL (211–911)
RBC	2.83 × 10⁶/μL (3.90–5.20)
HGB	101 g/L (120.0–156.0)
HCT	29.3% (35.5–45.5)
MCV	103.4 fL (80.0–99.0)
MCH	35.7 pg (27.0–33.5)
MCHC	345 g/L (315.0–360.0)
PLT	310 × 10³/μL (150.0–370.0)
WBC	5.2 × 10³/μL (3.5–10.0)
Neutrophils	2.8 (53.8%) (2.0–7.7)
Lymphocytes	1.9 (37.8%) (1.1–4.0)
Monocytes	0.38 (7.2%) (0.10–0.90)
Eosinophils	0 (0%) (0.02–0.75)
Basophils	0 (0.1%) (0.0–0.2)
Immature granulocytes	0.06 (1.10%) (0–0.04)
ESR	36 mm/h (0–20)
TSH	0.933 mIU/L (0.350–4.940)
25-OH Vitamin D	9.0 ng/mL (≥ 30.000)
CRP	16.410 mg/L (<5.0)
Creatinine	109 μmol/L (≥ 90.0)

**Table 2:** ANA Panel Results Bold values indicate positive results. ANA: anti-nuclear antibodies; dsDNA: double-stranded DNA; SS-A: Sjögren syndrome-related antigen A; Ro-52: a 52-kDa intracellular protein; SS-B: Sjögren syndrome-related antigen B; nRNP: nuclear ribonucleoprotein; Sm: Smith antigen; Mi-2 alpha: the chromodomain helicase DNA‑binding protein 3; Mi-2 beta: chromodomain-helicase-DNA-binding protein 4; Ku: Ku protein, part of the DNA-dependent protein kinase; CENP: centromere protein; Sp100: Speckled protein 100 kDa; PML: promyelocytic leukemia protein; Scl-70: topoisomerase I; PM-Scl: polymyositis-scleroderma; RP: RNA polymerase; Gp 210: glycoprotein 210; PCNA: proliferating cell nuclear antigen; DFS 70: dense fine speckled 70.

Antibody	Value
dsDNA	0
Nucleosomes	3
Histones	98
SS-A	131
Ro-52	158
SS-B	17
nRNP/Sm	2
Sm	4
Mi-2 alpha	2
Mi-2 beta	2
Ku	2
CENP A	1
CENP B	3
Sp100	3
PML	3
Scl-70	4
PM-Scl 100	2
PM-Scl 75	1
RP 11	2
RP 155	6
Gp 210	1
PCNA	1
DFS 70	2

Patients with atrophic gastritis may develop vitamin B12 deficiency due to the destruction of gastric parietal cells, leading to reduced production of intrinsic factor. In this patient, megaloblastic anemia was observed despite normal serum vitamin B12 levels, suggesting an early-stage or functional deficiency. Although vitamin B9 (folate) levels were within the normal range (not shown in Table 1), this occurs because the body has substantial hepatic stores of vitamin B12 that can last up to nine years before serum levels decline. These neurologic symptoms and hematologic findings developed later than her primary complaint of arthralgia.

One alarming symptom was sudden unilateral vision loss, attributable to systemic sclerosis via fibroproliferative vasculopathy. Ophthalmologic examination revealed acute retinal artery occlusion. No further diagnostic evaluation was pursued for the vision loss due to cost and ethical constraints. The patient has a history of secondary hypertension under cardiology care, which is well controlled, with no other abnormalities noted on further evaluation.

According to the American College of Rheumatology/European League Against Rheumatism (ACR/EULAR) classification criteria, our patient's symptoms/signs score above 9, which indicates the presence of SSc [12].

Two months after the initial rheumatology visit, her general status was much improved, with reduced joint and abdominal pain. Despite her unilateral vision loss, she developed a five-day history of urinary tract symptoms, such as dysuria, urgency, and back pain at the level of the kidney. Scleroderma renal crisis was considered but ruled out based on laboratory and imaging studies. The diagnosis was urinary tract infection, and antibiotics were prescribed.

Treatment included methylprednisolone 8 mg daily, methotrexate 17.5 mg weekly, folic acid, and vitamin C. Vitamin B12 intramuscular injections were initiated on the recommendation of the hematology specialist. On follow-up, her symptoms gradually improved, she felt more energized, and her quality of daily life was significantly enhanced.

## Discussion

Systemic sclerosis (SSc) is an immune-mediated rheumatic disease characterized by fibrosis of the skin and internal organs along with vasculopathy. Although uncommon, it carries significant morbidity and mortality [1]. Gastrointestinal involvement is common in systemic sclerosis in nearly 90% of patients. There are two main types of atrophic gastritis: Type A (autoimmune metaplastic atrophic gastritis, AMAG), which involves T-cell-mediated destruction of parietal cells and intrinsic factor, and Type B (environmental metaplastic atrophic gastritis, EMAG), which is limited to the antrum [2, 3, 5]. Autoimmune atrophic gastritis (AMAG) results from T-cell-mediated destruction of gastric parietal cells, leading to intrinsic factor deficiency and eventually megaloblastic anemia [2,4,5]. In this case, the patient developed megaloblastic anemia secondary to autoimmune atrophic gastritis despite normal serum vitamin B12 levels, highlighting the insidious nature of this complication [2,4]. The clinical and endoscopic findings were consistent with Type A autoimmune atrophic gastritis. All patients with atrophic gastritis should be tested for *H. pylori *and treated if positive [2, 5]. The presence of anti-Ro52 and anti-SS-A antibodies in this patient is consistent with the known association of these autoantibodies with gastrointestinal involvement in systemic sclerosis. However, autoimmune atrophic gastritis remains uncommon in patients with SSc [6]. 

Acute retinal artery occlusion, however, is an extremely rare and vision-threatening complication [7-9]. Ocular involvement occurs in approximately 50% of patients with systemic sclerosis, most commonly as dry eye or eyelid abnormalities [10]. The relatively protected microcirculation of the retina in systemic sclerosis makes this presentation particularly noteworthy [7, 8]. Histopathologic findings in such cases include retinal ischemic atrophy, concentric narrowing and fibrosis of small vessels, thinning of the retinal pigment epithelium and choroidal capillaries, ischemic areas with intraretinal extravasation and microaneurysms, and peripheral capillary non-perfusion [7-9].

To the best of our knowledge, this is one of the few reported cases of SSc presenting a 67-year-old female patient with two rare complications simultaneously: autoimmune atrophic gastritis with early megaloblastic anemia and acute retinal artery occlusion. This case is noteworthy because both complications occurred as prominent features, sometimes even preceding classic cutaneous manifestations. 

The management of such complex cases requires a multidisciplinary approach involving rheumatology, gastroenterology, and ophthalmology. Early immunosuppressive therapy, vitamin B12 supplementation, and vascular risk factor modification are essential. Regular screening for gastrointestinal and ocular complications may enable earlier detection and intervention.

Negative result of specific antibodies (Anti-Scl 70, Anti-centromere, and Anti-RNA polymerase-III) in serum does not exclude the diagnosis of Systemic sclerosis. If ANA is positive, the specialist should assume active autoimmune processes. Although the laboratory and other instrumental tests help us diagnose SSc, systemic sclerosis is mostly a clinical diagnosis. The patient's symptoms and signs help us to identify and diagnose SSc, especially with atypical manifestations. Negative serology test results and ignoring clinical manifestation may mislead the process of diagnosis and cause late or misdiagnosis of the disease [11]. In similar cases, ACR/EULAR classification criteria support specialist to diagnose SSc [12]. 

Patients with SSc and negative specific antibodies have the same treatment and management plan as seropositive SSc.

## Conclusions

Systemic sclerosis is a rare autoimmune disease characterized by fibroproliferative vasculopathy and progressive fibrosis that can affect multiple organ systems. Negative serology test result and clinical manifestations support the diagnosis of systemic sclerosis. Although gastrointestinal involvement is frequent, autoimmune atrophic gastritis with resultant vitamin B12 deficiency and acute retinal artery occlusion represent uncommon but significant complications.

This case illustrates the importance of thorough evaluation and a high index of suspicion for atypical manifestations in patients with systemic sclerosis and negative specific antibodies. Prompt diagnosis and multidisciplinary management are essential to improve patient outcomes and quality of life. Increased awareness of these rare presentations may facilitate earlier intervention and prevent irreversible damage such as severe anemia and vision loss.
